# Models and Methods to Investigate Acute Stress Responses in Cattle

**DOI:** 10.3390/ani5040411

**Published:** 2015-12-03

**Authors:** Yi Chen, Ryan Arsenault, Scott Napper, Philip Griebel

**Affiliations:** 1 Vaccine and Infectious Disease Organization, University of Saskatchewan, Saskatoon, SK S7N 5E3, Canada; E-Mails: yic575@mail.usask.ca (Y.C.); scott.napper@usask.ca (S.N.); 2 Department of Biochemistry, University of Saskatchewan, Saskatoon, SK S7N 5E5, Canada; 3 Department of Animal and Food Sciences, University of Delaware, Newark, DE 19716, USA; E-Mail: rja@udel.edu; 4 School of Public Health, University of Saskatchewan, Saskatoon, SK S7N 5E3, Canada

**Keywords:** bovine, cortisol, epinephrine, kinome, physical stress, psychological stress, immunometabolomics

## Abstract

There is a growing appreciation within the livestock industry and throughout society that animal stress is an important issue that must be addressed. With implications for animal health, well-being, and productivity, minimizing animal stress through improved animal management procedures and/or selective breeding is becoming a priority. Effective management of stress, however, depends on the ability to identify and quantify the effects of various stressors and determine if individual or combined stressors have distinct biological effects. Furthermore, it is critical to determine the duration of stress-induced biological effects if we are to understand how stress alters animal production and disease susceptibility. Common stress models used to evaluate both psychological and physical stressors in cattle are reviewed. We identify some of the major gaps in our knowledge regarding responses to specific stressors and propose more integrated methodologies and approaches to measuring these responses. These approaches are based on an increased knowledge of both the metabolic and immune effects of stress. Finally, we speculate on how these findings may impact animal agriculture, as well as the potential application of large animal models to understanding human stress.

## 1. Introduction

### Defining Stress

Early in the nineteenth century, Claude Bernard defined stress as a perturbation of the otherwise constant state of the “milieu interieur”. [1]. Fifty years later, Cannon redefined this concept using the word “homeostasis” [2]. Homeostasis was defined as “the coordinated physiological processes which maintain a steady state in the organism”. Cannon also proposed the “fight or flight response” as a major mechanism by which an organism responds to stress or danger [2]. Selye subsequently expanded the definition of stress to include, “the nonspecific response of the body to any demand made upon it” [3]. Selye defined three stages of the general adaptation syndrome (GAS) or biologic stress syndrome. The first stage is recognition of a noxious agent. The second stage is resistance or adapting the body to adjust to the stimuli, including release of secretory granules from the adrenal cortex into the blood stream that alter tissue metabolism. This stage is also recognized as the alarm reaction. The last stage, if stress persists, is the stage of exhaustion when acquired adaptation is lost and finally leads to disease [3]. This definition was important since it incorporated the element of time or duration to the stress response which is critical for differentiating between acute and chronic stress. In late twentieth century, McEwen and Goldstein considered stress as either a consciously or unconsciously sensed threat to homeostasis, which exists within the range of specific parameters [1]. This definition recognizes that the homeostatic state may shift over time and vary among individuals within a population.

These changing definitions reveal how difficult it is to precisely define stress responses, which now includes a much broader definition of the fight, flight or freeze response [4]. Contributing elements to stress responses have been defined as either psychological or physical factors that are perceived by an organism in a variety of ways. These factors disturb the homeostatic state, but the organism has the capacity to initiate complex and coordinated psychological and physiological responses to each stressor. These responses include not only the classic “fight or flight” response but also the “freeze” response, which may immobilize an animal [5]. Further, it is recognized that repeated exposure to stress may result in psychological changes that either dampen, exacerbate, or change the nature of the stress response. If stress is resolved then homeostasis can be restored but if a stressor persists or occurs repeatedly then perturbations of the homeostatic state may result in psychological and physiological pathology. From the perspective of domestic animal production, these pathologies may manifest as altered behavior, decreased immune protection that impacts disease susceptibility, or altered metabolism that impacts either growth, production, or a combination of these responses. Quantifying the impact of stress on animal production may be very difficult as many of these manifestations may occur at a subclinical level. Being able to measure the effects of stress on immunity and metabolism at a molecular level is critical if we are to engage in an informed discussion regarding the impact of a specific stressor, or combination of stressors, on animal health, well-being, and production. Furthermore, developing more accurate measures of stress will enable producers to monitor changes in animal husbandry or production systems and determine whether these changes reduce or eliminate the physiological effects of both acute and chronic stress.

## 2. The Stress Response

**Figure 1:**
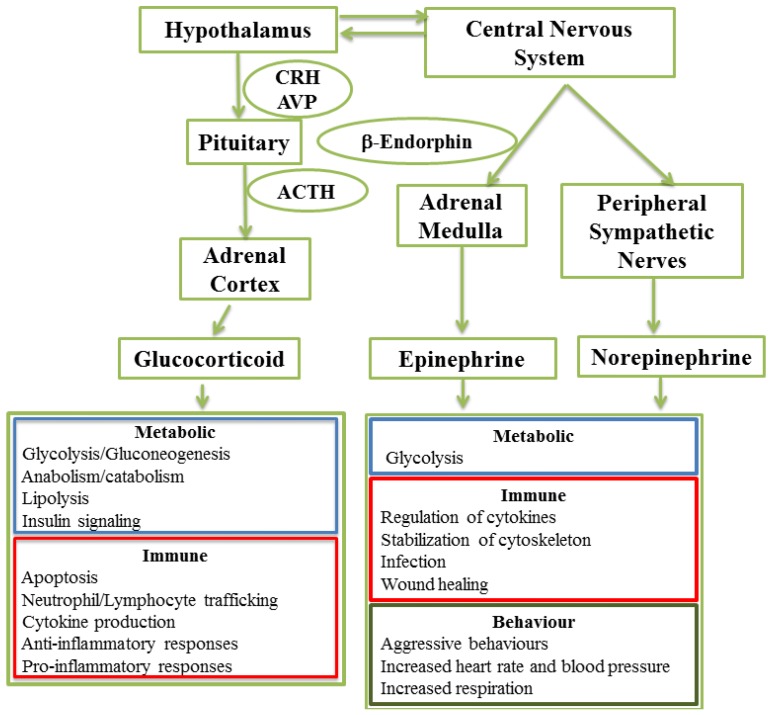
Biological functions regulated by the two stress axes. The hypothalamus-pituitary-adrenal (HPA) axis is activated when the body perceives a physical or psychological stressor. Corticotrophin releasing hormone (CRH) and arginine vasopressin (AVP) release from the hypothalamus results in adrenocorticotropic hormone (ACTH) secretion by the pituitary. ACTH then stimulates release of glucocorticoids from the adrenal cortex. The sympathetic-adrenal-medullary (SAM) axis is a coordinated response to diverse stressors mediated by the release of epinephrine and from the adrenal medulla and norepinephrine from peripheral sympathetic nerves. Cross-talk between the central nervous system and pituitary coordinate HPA and SAM axis activation and release of β-endorphin by the pituitary contributes to this cross-talk. Circulating glucocorticoids and catecholamines interact with a wide variety of cells to alter both metabolic and immune functions.

Two different signaling axes have been identified by which mammals mount an integrated physiological response to perceived danger (Figure 1). The response to danger is initiated at the level of the hypothalamus which releases corticotrophin releasing hormone (CRH) and vasopressin (VP) [6,7]. These hormones transmit a signal to the pituitary gland to initiate release of adrenocorticotropic hormone (ACTH) which targets the adrenal cortex. This hypothalamic-pituitary-adrenal (HPA) axis initiates one arm of the endocrine response to stress, mediated by the release of glucocorticoids from the adrenal cortex (Figure 1). The second arm of this response is very rapid and involves the sympathetic-adrenal-medullary (SAM) axis, which culminates in the release of catecholamines from the adrenal medulla (Figure 1). The SAM axis initiates the “fight or flight” response that includes an integrated behavioural response to perceived danger or acute stress as well as metabolic and immune responses [8,9,10] (Figure 1).

### 2.1. Glucocorticoid-Induced Signaling

Glucocorticoid receptors (GRs) are present in the cytoplasm of most cells and are low affinity receptors for cortisol. Following activation of the HPA axis, GRs are activated by the ensuing elevated concentration of cortisol [11]. Glucocorticoid receptors mediate signaling transduction by two mechanisms, including genomic and non-genomic actions [12]. Upon binding with cortisol, GRs become activated and disassociate from Heat Shock Protein 90 (Hsp 90) and Src complex. Activated GRs homodimerize and translocate from the cytosol into the nucleus where they can bind to Glucocorticoid response elements (GREs). GREs can either activate gene expression by recruiting cofactors and histone-modifying elements to the promoter region or GREs can repress gene expression by interacting with transcription proteins, such as nuclear factor κB (NF-κB), interferon regulatory factor-3 (IRF-3), or activator protein 1 (AP 1) [11]. GRs may also interact directly with membrane bound or cytosolic GRs and kinases, such as extracellular signal-regulated kinases (ERKs), c-Jun NH2-terminal kinases (JNKs), and p38 MAPK [12,13].

### 2.2. Metabolic Functions Activated by Glucocorticoid-Induced Signaling

Glucocorticoids and their receptors function as checkpoints for energy homeostasis and mediate many of the stress-related effects on metabolism. A recent study revealed that carbohydrate metabolism, including fructose and pentose phosphate pathways, was down-regulated in response to heat stress, resulting in decreased metabolism in bovine peripheral blood leukocytes [14]. Mehla and colleagues also reported altered expression of kinase genes involved in glycolysis and insulin-induced glucose uptake after heat stress treatment of cattle [15]. Further studies are required to determine whether these metabolic responses are unique to heat stress or are conserved for a variety of stressors. Glucocorticoid receptors also play an important role in regulating the balance between anabolism and catabolism. One isoform of glucocorticoid receptors, GRα has been shown to silence the expression of activating transcription factor 4 (ATF4), which is a transcription factor involved in amino acid biosynthetic enzymes, indicating a significant role for glucocorticoids in regulating anabolism and catabolism [16]. Lipoprotein lipase (LPL) activity was also reduced following glucocorticoid-induced lipolysis in adipose tissues [17]. Thus, glucocorticoids can regulate a broad range of metabolic functions.

### 2.3. Immune Responses Regulated by Glucocorticoid-Induced Signaling

Glucocorticoids can influence a broad range of both innate and acquired immune responses. For example, it is well known that glucocorticoids have anti-inflammatory effects, but a recent study demonstrated that glucocorticoids can also induce pro-inflammatory responses [12]. This is consistent with our recent study that determined combining maternal separation with transportation increased interferon (IFN)-γ secretion and enhanced acute-phase protein production following viral infection [18]. Glucocorticoids may also enhance innate immune responses by increasing the expression and signaling by Toll-like receptors (TLRs), such as TLR2 and TLR4 [12,19]. Griebel *et al.*, reported that individual stressors, such as transportation *versus* combined transportation and weaning, had significantly different effects when assaying lipopolysaccharide (LPS)-induced TLR4 signaling by bovine blood mononuclear cells [20]. The increase in LPS-induced secretion of tumor necrosis factor (TNF) was significantly greater in calves experiencing transportation alone than calves subjected to the combined stressor of transportation and abrupt weaning [20]. Thus, despite inducing a similar increase in serum cortisol, specific stressors may have significantly different effects on innate immune responses [18].

The expression of pro-inflammatory genes can be inhibited by glucocorticoids through an interaction of the GRs with transcription factors, such as NF-κB and AF-1 [21]. A variety of other regulatory proteins may also mediate the anti-inflammatory effects of gluococorticoids. For example, glucocorticoid inhibition of p38 mitogen-activated protein kinase (MAPK) was mediated by MAPK phosphatase-1 [22]. Contradictory results between the analysis of glucocorticoid effects *in vitro* and observations made following elevation of endogenous cortisol during a stress response suggest that the regulation of cell signaling may be much more complex *in vivo*. Unravelling this apparent contradiction may require new ways to analyze cell signaling responses initiated under the integrated influence of both the HPA and SAM axes.

Glucocorticoids have also been reported to have multiple effects on acquired immune responses (Figure 1). These effects may include altered lymphocyte trafficking, apoptosis of naïve lymphocytes, modulating cytokine secretion, decreasing the ratio of helper/suppressor T cells, and decreasing antibody productions [23]. The magnitude, duration, and timing of elevated cortisolemia required to effect a significant change in acquired immune responses remains to be determined. There are contradictory reports regarding the impact of stress on antibody responses in humans and mice following vaccination. Specific stressors either inhibited or enhanced antibody responses, depending on the strain of mice, the type of stressor, and the timing of stress relative to vaccination [24]. These observations indicate that it is difficult to predict how individual animals will respond and that each stressor, or combination of stressors, may have very different effects on an acquired immune response.

### 2.4. Catecholamine Signaling

Norepinephrine and epinephrine are two catecholamine neurotransmitters released by the SAM axis that exert effects through both α- and β-adrenergic receptors, but mainly β_2_- adrenergic receptors [9]. This interaction influences a wide variety of body systems, increasing heart rate, blood pressure, respiration rate, and decreasing activity of the gastro-intestinal tract. These integrated physiological changes are part of the “fight or flight” response observed following acute stressors [8]. At a cellular level, β-adrenergic receptors signal through G proteins that catalyze the synthesis of cAMP to activate cAMP-dependent protein kinase A (PKA) and transcription factors involved in regulating gene expression [25].

### 2.5. Metabolic Functions Activated by Catecholamine-Induced Signaling

Epinephrine influences glucose metabolism through two mechanisms. Firstly, through protein kinase AMPK-mediated activation of the transcriptional factor, coactivator peroxisome proliferator-activated receptor-γ coactivator 1α, which stimulates mitochondrial biogenesis. This results in an increased capacity for glucose metabolism [26]. The other mechanism is that insulin and AMPK together increase the glucose transporter, GLUT4, and translocate GLUT4 to the cell membrane resulting in increased cellular uptake of glucose [26]. Catecholamines may also influence insulin signaling [27], lipid metabolism [28], and thermogenesis [29].

### 2.6. Immune Responses Regulated by Catecholamine-Induced Signaling

Catecholamines have also been linked to alterations in a wide range of immune functions (Figure 1). Epinephrine has been reported to inhibit wound epithelialization [30], alter neutrophil trafficking [31], regulate cytokine secretion [32], and change host–pathogen interactions by altering microbial growth [33]. Treatment with reserpine, an indole alkyloid, reduces the level of circulating norepinephrine and increases the level of TNF-α. Further, increasing the concentration of circulating norepinephrine decreased LPS-induced TNF-α responses in mice [32]. These observations support the conclusion that circulating catecholamines play a role in regulating cytokine production [32]. Epinephrine released from karatinocytes, can also act in an autocrine fashion to bind β_2_ adrenergic receptor and induce activation of AKT signaling pathways. This signaling stabilizes the cytoskeleton, increases focal adhesion formation, and eventually inhibits karatinocyte migration. These epinephrine induced changes in epithelial cell function can result in impaired wound healing [30].

## 3. Stressors Studied in Cattle

**Figure 2:**
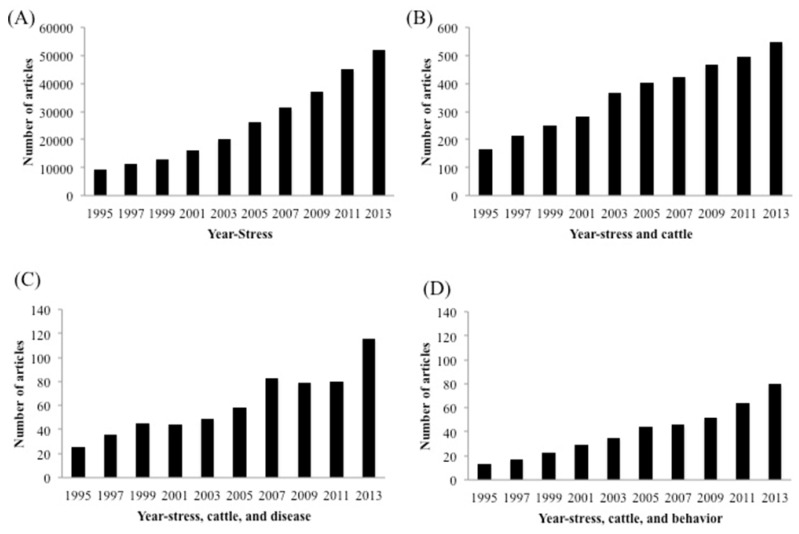
Number of publication entries in Medline (PubMed) trend * from 1995 to 2013. (**A**) Publication entries searched with query “stress”; (**B**) Publication entries searched with query “stress and cattle”; (**C**) Publication entries searched with query “stress, cattle, and disease”; (**D**) Publication entries searched with query “stress, cattle, and behavior”. * Medline (PubMed) trend URL: http://dan.corlan.net/medline-trend.html.

**Figure 3:**
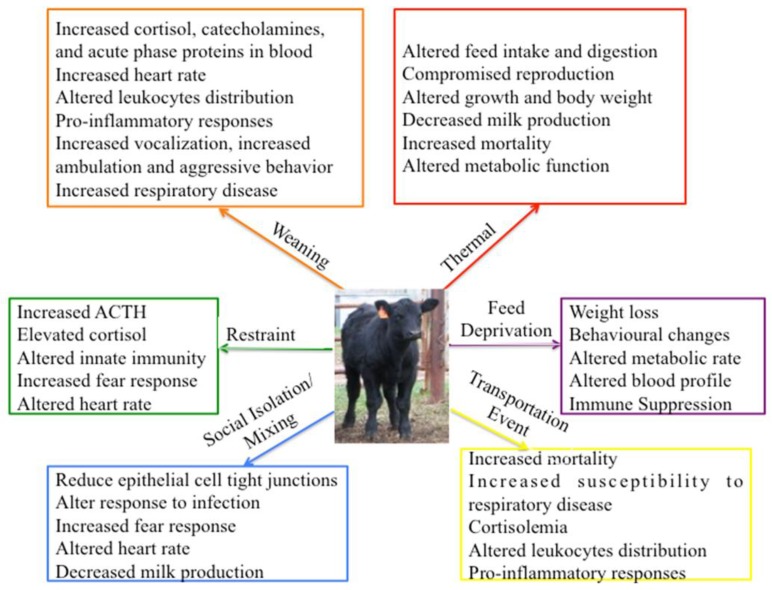
Biological functions altered in response to individual stressors. For each stressor investigated in cattle a variety of functions were investigated and evidence presented to demonstrate significant biological or psychological changes. The results from individual studies may be confounded by the presence of one more stressors.

Research related to stress in all species has increased dramatically over the last 20 years with over 50,000 citations in 2013 (Figure 2A). In contrast, research activity related to stress in cattle has remained relatively static with fewer than 500 citations/year over the last 4 years, representing less than 1% of all stress-related research (Figure 2B). Research related to stress and disease represents almost 25% of current bovine stress-related research (Figure 2C) but research related to stress and behavior represents less than 10% of bovine stress-related research (Figure 2D). Numerous stressors have been investigated in cattle over the last 50 years and these stressors can be divided into either physical or psychological stressors (Table 1). These are convenient categories for the classification of stress but cattle experience many stressors concurrently during normal animal husbandry practices. This complicates the investigation of possible links between stress and decreased animal production or health. Researchers attempt to simplify this situation by subjecting animals to a single controlled stress, but these studies may be complicated by stress effects related to monitoring animals during the study. We will review the range of stressors and model systems used to investigate the biological effects of stress in cattle (Figure 3) but acknowledge that many studies were complicated by exposure to multiple stressors, including effects related to sample collection.

**Table 1:** Physical and psychological stressors investigated in animal.

Stressor	Cattle	Pigs	Hens	Sheep	Duck	Horse	Mice	Rats
**Physical stressors**								
Thermal Stressors								
Cold	✔	✔	✔	✔	✔	✔	✔	✔
Heat	✔	✔	✔	✔	✔	✔	✔	✔
Transportation	✔	✔	✔	✔	✗	✔	✔	✔
Feed Deprivation/restriction	✔	✔	✔	✔	✔	✔	✔	✔
Noise	✔	✔	✔	✗	✗	✗	✔	✔
**Psychological stressors**								
Weaning	✔	✔	✗	✔	✗	✔	✔	✔
Social isolation/mixing	✔	✔	✔	✔	✔	✔	✔	✔
Restraint	✔	✔	✔	✔	✔	✔	✔	✔

### 3.1. Physical Stressors

#### 3.1.1. Thermal Stress

Thermal stressors, including both heat and cold stress, are commonly experienced by livestock living in a natural environment. Heat stress has been studied in dairy cows and a variety of outcomes have been documented, including negative consequences for both health and productivity (Figure 3). Heat stress was associated with a reduction in both milk production [34] and body weight [35]. The most severe outcome of heat stress is mortality but heat stress for one to three weeks during late term pregnancy can also affect newborn calves. Calves born to heat-stressed cows were observed to have a significantly shorter gestation, lower body weight, reduced absorption of colostrum, and changes in blood leukocyte function, phenotype and gene expression [36,37]. Understanding the mechanisms by which heat stress induces physiological and biological responses will be beneficial for improving animal health and productivity [38,39]. Gene expression in peripheral blood leukocytes was analyzed following a 4 h heat stress period with heat stress confirmed by measuring elevated skin temperature and respiration rates [14]. Microarray analysis revealed heat stress had a significant impact on carbohydrate metabolism, such as glycolysis and gluconeogenesis, and also altered expression of immune response genes, including those associated with the TLR and NOD-like receptor signaling pathways. It remains to be determined, however, if heat-induced changes in gene transcription translate into a significant change at the protein level. The effect of cold stress on cattle has not been extensively investigated but cold stress has been reported to cause changes in appetite, digestive function, metabolic function, and death [40]. Further research is required in this area.

#### 3.1.2. Transportation Stress

Transportation is one of the most common stressors experienced by cattle, especially with increased national and international movement of animals. Epidemiological studies have implicated transportation as a factor contributing to increased disease susceptibility and bovine respiratory disease for over 50 years [41,42]. A frequent observation following transportation is a transient increase in blood cortisol concentration (Figure 2). Many factors may contribute to this stress response, including human–animal interactions immediately prior to and after transportation [43]. Associated with this cortisolemia is an increased neutrophil to lymphocyte ratio [44]. More recently, it has been suggested that monitoring natural killer (NK) cell numbers in blood may provide a more specific measure of transportation stress responses than differential leukocyte counts [45]. Ship transportation is commonly used for the international movement of livestock and may represent a much more prolonged and complex stressor. Sea journeys may expose animals to a combination of increased noise, elevated temperatures, and high ammonia concentrations. These factors have been associated with increased alkalosis, pulmonary inflammation and mucosal irritation, and an increased incidence of respiratory disease [46]. The complex combination of stressors associated with transportation events have been associated with a broad range of physiological responses, including altered immune function, behavioral responses, and changes in muscle physiology [47]. These diverse responses highlight the potential for complex interactions among multiple stressors with each combination of stressors resulting in a unique physiological response.

#### 3.1.3. Feed Deprivation

Feed deprivation may occur at many levels, involving specific micronutrients or inadequate intake of protein or energy. Controlled protein-energy malnourishment over a 4 week period in newborn Holstein calves resulted in altered blood leukocyte counts, decreased lymphocyte function, and reduced antibody responses [48]. Holstein cattle subjected to controlled feed deprivation displayed both behavioral and physiological responses [49]. Behavioral changes included an increased reactivity to stressful events and physiological responses included elevated plasma cortisol levels and decreased β-hydroxybutyrate levels. Feed deprivation effects on rumen microflora and rumen function have also been investigated [50]. Rumen pH increased significantly during a 32-h period in feed-deprived steers and there was a concurrent decrease in rumen bacteria and protozoa [50]. These controlled feed deprivation experiments demonstrate that malnourishment may have profound effects, especially in young growing animals or lactating cows that have a high metabolic demand. It is also interesting to note, however, that even relatively brief periods of feed deprivation may have a pronounced effect on the gut microbiome. The importance of this observation is highlighted by recent evidence that a gut-microbiome-brain axis exists and that changes in the gut microbiome may have a direct impact on the HPA axis [51]. Therefore, future analyses of stress, including not only feed deprivation, may require a detailed profiling of the gut microbiome. These types of studies could determine if alterations in the gut microbiome contribute to stress responses and the increased disease susceptibility associated with important dietary transition periods, such as weaning of young calves.

### 3.2. Psychological Stressors

#### 3.2.1. Maternal Separation and Weaning

Maternal separation has been identified as a potent stressor in infants and young animals that may have long-term psychological and physical effects [52]. Abrupt weaning or separation of suckling calves from their dams is a common husbandry practice in both the beef and dairy industries. In 5 to 6-month old beef calves, abrupt maternal separation results in both the psychological stress of breaking the maternal bond as well as nutritional changes associated with dietary changes [53]. This separation results in behavioural changes in both calves and cows that may persist for several days, consistent with a more chronic form of stress. For calves, behavioural changes include increased vocalization and ambulation and this activity persists at an elevated level for at least three days after separation [53]. Lefcourt and Elsasser observed a significant increase in norepinephrine and epinephrine concentrations at 24 h after calves were separated from their dams but serum cortisol concentrations did not change significantly [54]. Re-uniting cows and calves resulted in a rapid decline in norepinephrine and epinephrine concentrations [54]. Studies in rodents have demonstrated that early maternal separation can have long-term effects on the gut-microbiome-brain axis and stress responses adult animals [55]. The long-term consequences of maternal separation in newborn and older calves have yet to be investigated.

The effect of calf age at the time of weaning on innate immune responses of beef calves has been studied. An endotoxin challenge study revealed that following weaning, 80-day old calves had lower TNF, IL-1, and IL-6 responses than 250-day old calves [56]. In contrast, interferon (IFN) and acute-phase protein responses were greater in early weaned calves than normally weaned calves [56]. It was concluded that there were age-dependent differences in the capacity of calves to recognize and respond to endotoxin. These authors did not, however, quantify stress responses to maternal separation to determine if these may have contributed to the age-dependent difference in innate immune responses. It is also possible that earlier weaning may be associated with more profound perturbations in the gut microflora when calves are more dependent on milk as a source of nutrition. It is important to differentiate between the psychological effects of the maternal bond and the dependence on maternal nutrition. Haley *et al.*, used nose-paddles to differentiate between a calf’s dependence on maternal nutrition and the psychological bond with a mother. These studies demonstrated that dependence on maternal nutrition was an important factor contributing to the psychological response following maternal separation [53]. This model system may provide a useful tool for future studies focusing on the gut microbiome and the role of the gut-microbiome-brain axis in stress responses.

#### 3.2.2. Social Isolation and Mixing

Cattle are herd animals that establish social orders with dominant and submissive animals within each group. As such, social isolation or introduction to a new social group can be significant stressors for an individual animal [57] resulting in diverse behavioral and physical responses (Figure 3). Ninomiya and Sato evaluated the effects of social isolation stress by measuring salivary cortisol and chromogranin A-like immunoreactivity (IR) to reflect HPA activity and SAM activity, respectively [58]. Chromogranin A was significantly elevated in socially isolated cows but cortisol concentrations remained unchanged two hours after social isolation [58]. The effect of prolonged social isolation was examined in Friesian cows isolated for a period of either 4 or 8 weeks. Socially isolated cows displayed behavioral changes with increased self-grooming and leaning when compared to control cows but no differences in serum cortisol concentrations were observed [59]. Thus, social stressors may not engage both the HPA and SAM axes and behavioral adaptations may occur in response to this type of stressor.

The effect of social isolation on physiological parameters has also been investigated using a respiratory disease model. Veal calves infected with bovine herpesvirus 1 (BHV-1) were randomly divided into two groups. One group was housed as individual animals while the other group was housed collectively. Socially isolated animals had lower basal cortisol concentrations and responded to ACTH injection with a decreased cortisol release relative to group-housed animals [60]. These studies suggest that social isolation reduced the severity of clinical disease but this observation may have been compounded by a reduction in viral transmission when animals were housed individually. A more direct link between serum cortisol and physiological responses to social isolation stress was observed by using serum lactose concentrations as a measure of epithelial cell permeability in the mammary gland [61]. Cows were first divided into high serum cortisol *versus* low serum cortisol groups based on measurements during their first lactation. When cows from both groups were then socially isolated for 55 h, epithelial cell permeability was consistently greater in the high cortisol group. The authors concluded that social isolation altered epithelial tight junctions and mammary gland function. Collectively, these observations indicate that social isolation not only alters behavioral responses but may also have an impact on a wide variety of body systems.

Current husbandry practices frequently result in social mixing or regrouping of both young and old cattle. A number of studies have analyzed acute behavioral and biological responses following the introduction of a single animal to an established group. In dairy cows, the introduced animal displayed altered behavior, including reduced allogrooming and decreased feed access, and this was associated with a 24 h reduction in milk production [62]. Similarly, the introduction of a single, milk-fed dairy calf to an established group resulted in transient changes in behavior and decreased feed consumption that lasted for a single day [63]. In contrast, co-mingling two groups of 8 dairy cows resulted in behavioral changes and decreased milk production that persisted in all animals for weeks [64]. Veissier and colleagues investigated the effect of repeated regrouping on pairs of male Holstein calves. They observed that calves appeared to habituate to repeated re-grouping but did display an increased adrenal cortex sensitivity to ACTH stimulation [65]. Thus, both young and old animals may adapt quickly to social mixing when a single animal is introduced to an established group. In contrast, the co-mingling of larger groups may have much longer term effects on both behavior and physiology, representing a chronic stress response.

#### 3.2.3. Restraint Stress

Restraint is a common procedure used when handling cattle and may have negative effects on productivity [66]. Herskin *et al.*, investigated the combined effect of restraint and social isolation on adrenocortical and nociceptive responses in dairy cows [67]. Plasma cortisol concentrations did not, however, differ between restrained and control cows and it was postulated that either the nociceptive stimulus or the 15 minute restraint procedure were not sufficiently robust to induce a stress response [67]. Szenci *et al.*, studied the effect of restraint stress in pregnant heifers [68]. Plasma cortisol concentrations were significantly elevated after restraint in a crush for 2 h on two consecutive days. However, when the restraint was repeated on the third day, there was no significant increase in plasma cortisol concentrations. These observations support the conclusion that cattle can rapidly adapt to some types of repeated stress [68].

Andrade *et al.*, also investigated the effect of repeated restraint episodes in Brahman cattle. Entry order, serum cortisol concentrations, heart rate, and respiratory rate were recorded during the experiment [66]. In addition, a subsequent experiment 6 months later was conducted with a mask placed over the face of the cattle. Cattle were restrained in a squeeze chute for 10 min each day over a 19 day period and plasma cortisol concentrations gradually declined over the 19 day period [66]. These results are consistent with Szenci *et al.*, [68], reflecting habituation of animals to handling and restraint. Furthermore, placing a mask over the face of animals during restraint significantly reduced cortisol concentrations, heart rates, and respiratory rates. Thus, visual information appeared to be an important factor contributing to the stress response. Interestingly, Andrade *et al.*, also reported that the order in which calves were willing to enter the chute was consistent among experiments [66]. Thus, the stress response to restraint may begin prior to confinement. Cellular signaling pathways mediating physiological changes in response to restraint and the duration of responses following release from restraint have not been well characterized [69].

#### 3.2.4. Stress and Disease

Epidemiological evidence has linked a wide variety of stressors, especially transportation, with respiratory disease which was referred to historically as “shipping fever” [70]. Transportation may, however, be comprised of many different stressors, including feed and water deprivation, thermal, restraint, and social re-organization during and after transfer to a new environment. Furthermore, commingling animals from multiple sources during this process may increase exposure to viral and bacterial agents [71]. Thus, it has been difficult to quantify the impact of stress on disease susceptibility. Using an experimental respiratory disease model, based on a combined viral and bacterial respiratory infection, Hodgson *et al.*, were able to demonstrate that weaning and transportation doubled mortality in 5 to 6-month old, Angus X Hereford calves. Increased mortality was associated with a significantly enhanced innate immune response to both the viral and bacterial infection. This enhanced innate immune responsiveness also resulted in a significantly decreased interval between infection and death, suggesting increased immunity resulted in immune pathology rather than immune protection [18]. This is consistent with previous reports that glucocorticoids may increase pro-inflammatory responses [12,19]. A link between stress and an increased susceptibility to infectious diseases such as mastitis [72], Johne’s disease [71], and salmonellosis [73] in dairy cows has been reported. This area of research has increased greatly in the last few years (Figure 2C). It is difficult, however, to validate these observations unless animals are subjected to controlled stressors and then challenged with a specific pathogen or monitored for specific metabolic changes. These controlled studies are required if we are to quantify the contribution individual or multiple stressors make to reduced disease resistance or loss of production.

## 4. Measuring Stress Responses

Quantifying responses to experimental or natural stressors is an important first step in determining the potential effects of acute or chronic stressors. Developing tools that can measure complex physiological or psychological responses is also important if we are to begin unravelling the interactions among multiple stressors, which may include both psychological and physical stressors. We have observed that transportation alone has markedly different effects on innate immunity than a combination of weaning and transportation [20]. The differences noted were limited by the tools used to measure innate immunity and a much more comprehensive transcriptomic or proteomic analysis would certainly reveal more profound biological differences.

### 4.1. Measurement of Behavioral Responses to Stress

Behavioural responses to stress have been well studied in mice but a limited number of stress-induced behavioural studies have been performed in cattle (Figure 2D; Table 2). Entry order into a chute system has been used as one parameter to measure the relationship between anxiety-related behavior and activation of the HPA axis. Bristow and Holmes reported higher serum cortisol concentrations correlated with delayed entry into a chute and Andrade *et al.*, reported entry orders were consistent across replicate restraint experiments [66,74]. These observations suggest that either prior conditioning or genetics may contribute to this anxiety-related behavior and activation of the HPA axis. Curley and colleagues also demonstrated that exit velocity following restraint in a chute can provide a quantitative measurement of temperament that correlated with serum cortisol concentrations [75]. Another behavioral measurement, chute score, has been used as a subjective measurement of behavioral responses to restraint. Chute score measurements provided a semi-quantitative measure, using a graded scale to score both the time an animal spent struggling and the vigor with which an animal struggled [76]. Frequency of vocalization and the distance animals walked or time spent recumbent have also provided quantitative measures of psychological stressors over an extended time period [53,74,77]. Thus, while several parameters have been successfully used to assess behavioral responses to stress there is substantially more work required to integrate these behavioral responses with physiological changes.

**Table 2:** Behavioral responses measured in beef cattle exposed to stress.

Behavioral Responses to Stress	Representative Reference
Entry Order	[74]
Chute Scores	[75]
Pen Scores	[75]
Exit Velocity	[75]
Vocalization	[53,74]
Recumbancy/walking	[53,77]
Rumination	[74]
Rope pulling	[78]

### 4.2. Measuring Physiological Responses to Stress

Activation of the HPA axis is frequently monitored through measurement of cortisol concentrations in either the plasma or serum [6], but the rapid and pulsatile release of corticosteroids from the adrenal cortex make this a very dynamic response. To minimize this problem blood samples are generally collected within minutes of an animal being exposed to the stressor [7], but cortisol concentrations may remain elevated for days when animals are responding to a sustained stress [18]. Cattle have a basal cortisol concentration of 15–25 nmol/L which can rapidly increase to 60 to 200 nmol/L, depending on the stressor and individual animal responses [7]. Further, the release of endogenous cortisol displays a circadian rhythm, with peak levels usually occurring in the morning [7]. Thus, it is critical that both the method of sample collection and the time of samples collection be considered when designing stress experiments and interpreting data.

Cortisol concentrations in cattle have also been measured in urine, saliva, hair, feces, and milk (Table 3). Collection of these body fluids may be less invasive than venipuncture and may, therefore, reduce the effects of sample collection. The measurement of cortisol levels in hair has been explored as a method to monitor stress responses over a more extended time period and minimize the effect of fluctuations due to circadian rhythm, seasonal changes, and animal handling [79]. Only one study has reported an association between bovine hair cortisol and serum cortisol concentrations following repeated ACTH injections [80]. This association may be complicated, however, since cortisol may enter hair either from blood or through local cortisol production in the hair follicle [81,82]. Hair cortisol concentrations may also vary depending on the collection site, hair color, the sex and age of animals and the methods used to process hair samples [79,80]. While sensitive and standardized assays are available to measure cortisol levels in a wide range of biological samples, there remain substantial concerns regarding the limitations of sample collection, processing, and data interpretation. Commercial enzyme-linked immunosorbent assays (ELISAs) are now available to quantify epinephrine and norepinephrine concentrations in plasma but they require extraction procedures and the kits are expensive.

HPA and SAM axes activity has been measured by monitoring a diverse range of biological responses in cattle (Table 3). For example, total leukocytes and the neutrophil:lymphocyte ratio in blood has been used to measure the effects of cortisol on leukocyte trafficking [83,84]. Heart rate and respiratory rate [66] have been used as possible indicators of SAM activity (Table 3). Monitoring metabolic activity through changes in body temperature or weight and the abundance of metabolites have also been used as measures of stress responses (Table 3). Changes in innate immune responses or inflammatory mediators have also provided indirect measures for a variety of stress responses (Table 3) but each of these measurements provides insight into only one facet of a very complex biological response. Transcriptional profiling of gene expression in blood leukocytes and proteomic profiling of serum proteins have also been used to provide a broader picture of these responses and have revealed how dynamic these responses may be over time [8]. Thus, it has been difficult to select a single biological response or parameter that can be effectively used to quantify a stress response or discriminate between animals that respond differently to the same stressor. Discrimination among animals that differ in their exposure to stress or their response to a stressor is possible, however, when using an integrated analysis of multiple parameters, such as serum proteins, over the course of time [8]. A more comprehensive analysis of multiple physiological responses may provide an effective strategy to begin determining whether different stressors, either alone or in combination, have distinct biological effects that may be additive or synergistic.

**Table 3:** Parameters used to measure physiological reponses to stress in cattle.

Physiological Responses to Stress	Reference
Cortisol Measurement	
Serum/Plasma	[74]
Urine	[85]
Salivary	[86]
Hair	[80]
Milk	[87]
Epinephrine	[54]
Norepinephrine	[54]
Faecal metabolites	[88]
White Blood Cell Counts	
Complete Blood Count	[45]
Neutrophil/Lymphocyte	[45]
Heart rate	[66]
Respiratory rate	[66]
Body Temperature	[14]
Glucose	[89]
Pyruvate	[15]
Acute Phase Proteins	[77]
Innate Immune Responses	[90]
Body Weight	[77]
Serum proteome	[8]
Transcriptional Profiles	[14]

## 5. Immunometabolism

The integrated study of the immune system and metabolism is an emerging research perspective. Historically, immunity and metabolism were treated as distinct biological processes. Immunity focuses on the recognition of and resistance to a pathogen or toxin and involves its own set of cells and tissues. Metabolism incorporates chemical processes that provide the energy to carry out the various functions of the organism. Energy-dependent functions include both innate and acquired immune responses. However, the overlap between immunity and metabolism has mostly been restricted to metabolism as a source of energy for the immune system. In view of the many effects of both the HPA and SAM axis on metabolism, it is then important to consider in greater detail the potential interaction between immunity and metabolism.

**Figure 4:**
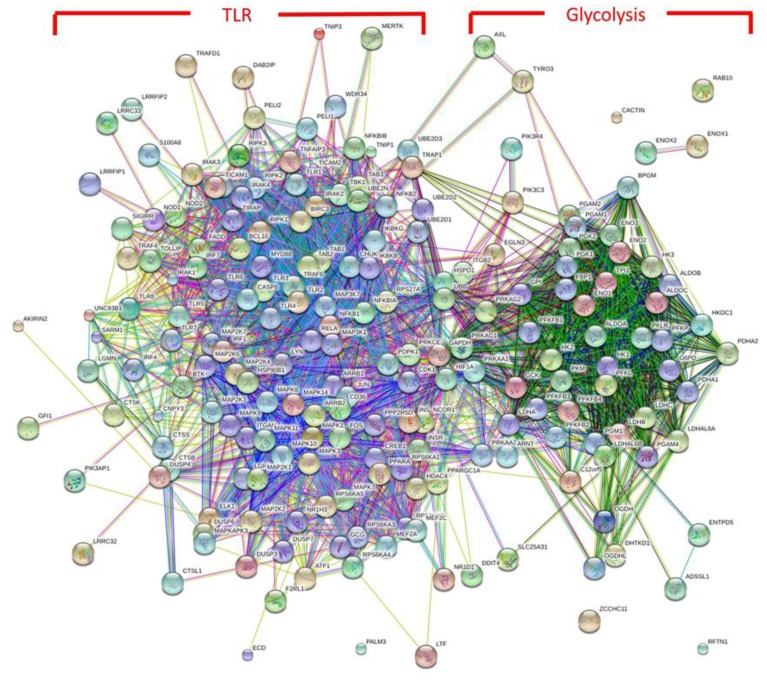
Protein–Protein Interaction Network. A list of proteins involved in glycolysis and toll-like receptor signaling was input into the STRING protein–protein interaction database. The cluster to the right represents protein interactions known to be involved in glycolysis. The cluster to the left represents proteins known to be involved in TLR signaling. Extensive protein interactions can be observed between the two signaling clusters.

The links between metabolism and immunity are extensive, and new interactions continue to be discovered. Figure 4 illustrates the known overlap in protein–protein interactions involved in glycolysis and TLR signaling. Two distinct protein interaction clusters can be observed for glycolysis and TLR signaling, but a significant number of common protein interactions are apparent between these two signaling nodes. The figure, based on using currently available data, provides a visual representation of immunometabolism and how links or interactions between these two biological processes occur. The challenge is to develop high-throughput methodologies that can analyze relevant protein–protein interactions and provide a more detailed picture of physiological responses to stress. These detailed immunometabolic profiles will not only provide insight into the mechanisms by which stress alters the homeostatic state but by using this more detailed picture it may be possible for the first time to develop biological profiles for individual stressors or combinations of stressors.

It is apparent that the responses of animals to different stress challenges are complex and multifaceted with unique phenotypic outcomes. To better understand these outcomes there is a need to better understand the molecular mechanisms that underlie stress responses. As already highlighted in this review, a number of investigative approaches, focused on different aspects and levels of molecular response have been employed to elucidate key events underscoring either metabolic or immune responses to stress. One emerging approach that is showing particular promise to provide a more comprehensive view of both metabolic and immune functions is the analysis of signal transduction at the level of kinase-mediated phosphorylation-dependent signal transduction. Across all eukaryotic species, protein phosphorylation is a central mechanism to regulate virtually every cellular process. These phosphorylation reactions are catalyzed by kinases and the collective activity of all the cellular kinases is referred to as the kinome.

Protein phosphorylation mediated by kinases often represent the event initiating activation of a cellular process following the receptor–ligand interactions. This is of particular relevance when considering the release of mediators following activation of either the HPA or SAM axis. Understanding cellular responses at the level of the kinome offers an advantage over other omics approaches by providing insight at a biological level that immediately precedes phenotypic changes. As such, kinome analysis has the potential to offer a clear and unobstructed view of critical cellular responses while transcriptomics reflect cellular responses induced by this initial signaling. Kinome analysis is a relatively new technique but one of the first applications was to understand signaling responses induced in blood leukocytes of human volunteers immediately following an acute stress [91]. From this initial investigation the power of this approach to offer predictive insight into complex biological responses was apparent. In particular this example highlighted the potential of the technology to contribute to a molecular understanding of stress responses within the context of a model of acute stress.

One challenge that often faces livestock researchers is the availability of new technologies that can be readily translated to the species of interest. A lack of cutting edge tools and reagents often limits the extent to which biological responses can be defined within food-producing animals. Fortunately, a variety of species-specific peptide arrays have been developed for kinome analysis. Arrays have been developed for cattle [92,93,94], pigs [95], chickens [96], turkeys [97] and honeybees [98]. This includes the development and application of arrays that are not only customized towards species, but also towards biological responses. A priority of these new generation arrays has been towards understanding the responses of livestock to stress with particular emphasis of signaling events associated with immunity and metabolism. Thus, the kinome tools are now available to begin using established models of acute and persistent stress (Table 1) to generate integrated metabolic and immune response profiles. A subsequent challenge will then be to analyze these large data sets to provide distinct profiles that can effectively discriminate between different types of stressors and identify animals that better tolerate or adapt to each stressor.

## 6. Implications

Stress has become a central issue in the growing debate on animal welfare and the psychological and physiological responses of animals to traditional husbandry procedures and new production technologies [99]. This debate has many perspectives that include special interest groups, consumers, and producers. Furthermore, the debate must address important issues regarding food safety and quality, animal health and production, and the sustainability of current cattle production practices. Many of these issues are highly emotive and for an effective discussion it is important the debate be informed by scientific data that can provide quantitative measures of the magnitude and duration of stress responses in animals.

### 6.1. Changing Approaches to Animal Agriculture

The management of pain and the stress associated with surgical manipulations, such as castration and dehorning, highlights the increasing concern regarding animal welfare [100]. New technologies, such as immunocastration, are being tested to circumvent surgical procedures and the breeding of polled cattle eliminated the need for dehorning. It is much more difficult, however, to eliminate many of the other common sources of stress, such as the social mixing and restrain of animals in a chute when administering vaccines or other therapeutic compounds. Furthermore, the selection of animals for enhanced production traits, such as meat or milk production, has placed an even greater metabolic demand on these animals. The emergence of immuometabolomics makes it even more apparent that selecting animals for increased production has direct implications for immune function. This convergence of metabolic and immune function is also of direct relevance when considering the highly coordinated physiological response to stress.

The co-evolution of domestic animals with humans has changed many of their behaviours and possibly their perception of danger or stress. If we can apply integrated tools, such as kinome analysis, to the measurement of physiological responses to stress it may be possible to begin the selection of animals with reduced stress responses. This integrated analysis of physiological responses will be important to not only understand the mechanisms mediating stress responses but to also understand the biological consequences of a reduced or altered stress response. Cannon defined stress as “the coordinated physiological processes which maintain a steady state in the organism” [2]. Therefore, the most effective strategy may be to select animals that rapidly adapt to new or changing situations while understanding both the metabolic and immune costs associated with this adaptation. Understanding these biological costs may provide a more rational approach to the selection and production of animals that provides an optimal balance between health and production.

### 6.2. Translation to Human Health

Anxiety and stress-related disorders are debilitating psychiatric conditions that affect health, wellbeing and productivity of a large percentage of the population [101,102]. Anxiety disorders are the most common mental illness in the US, affecting an estimated 40 million adults at an estimated economic cost in the 1990s of 42 billion dollars each year [103]. Despite the importance, and therapeutic attention, given to stress-related illnesses, the diagnosis and treatment of stress is an inexact and inefficient process [104]. Currently, the establishment of effective medication regimes for individuals with stress-related disorders is often a trial-and-error process based on self-reported patient feedback to various treatment regimes.

Among the many challenges to effectively diagnosing and treating stress is the wide spectrum of root causes and variation in individual’s responses to stress. The identification of molecular markers that effectively discriminate among various stresses, as well as adaptive and pathological responses to these states, would be of enormous benefit. For example, saliva cortisol levels are being examined as an indicator of various types of stress disorders as well as a read-out to monitor the efficacy of different treatment approaches [105]. While an effective rationale, cortisol concentrations provide only a crude assessment of the complexity and diversity of biological stress. More sophisticated and discriminating markers could help guide more accurate diagnoses of stress, inform rationale approaches for therapeutic intervention and provide a mechanism to monitor the success of treatment. Establishing better defined animal models of stress may help address these issues by providing a better understanding of the biological responses to individual or combined stressors and the signaling pathways that mediate these responses at a cellular level. Identifying specific signaling pathways may then provide quantifiable biomarkers of various stress disorders.

Currently, as is the case for most animal research, rodents are the primary species for investigating stress responses and therapeutics. Mice offer considerable advantage in terms of cost, ease of handling, defined tools and tests for measuring cognitive responses, and availability of genetically manipulated mice to model human diseases. There are, however, growing concerns over the efficiency with which results obtained from mouse models can be translated to clinical application in humans. In particular, Seok *et al.* demonstrated that the vast majority of research relating to immunological responses of mice did not have consistent results within human systems [106]. This finding is important from the broader perspective of highlighting biological differences between humans and mice, differences that are likely to manifest in a variety biological contexts, including stress. More specifically, as immunological responses and capabilities are strongly impacted by stress, this casts further doubt on the capacity of mouse models to accurately anticipate and reflect human responses to stress. To address these limitations of the mouse model, we suggest that greater consideration of large animal species might be of considerable benefit.

The first advantage of a comparative approach to understanding stress responses across a number of species will be a greater evolutionary perspective on stress and the extent to which various responses are conserved across mammalian species. Understanding the conserved, core biology of stress responses, as well as unique species-specific differences, will help guide the selection of animal models based on conservation of molecular responses to similar stressors in humans. This will improve both the interpretation of results and their translation across species. The selection of animal models that best reflect or mimic human molecular responses would provide increased confidence in the selection or testing of therapeutics.

Secondly, while research in mice enhances reproducibility by controlling genetic diversity, this does not accurately reflect the diversity of biological responses among humans to particular situations and/or treatments. A defining feature of many large animal experiments is the extent to which responses to a stimulus vary among individuals within an outbred population. While this complicates experiment design and interpretation, this provides a more accurate perspective on the challenges faced during the translation of results to clinical application. Furthermore, understanding the mechanisms mediating biological responses at the extremes of a specific phenotype is of central importance for defining biomarkers and treatments within human populations where these extremes are likely to represent priority patients. Finally, appropriately selected large animal models may be benefit in the identification of treatments that more effectively translate into human therapies. There are increasing examples of the use of large animal models, such as pigs, for the testing of therapeutics in the contexts of infectious disease [107], cancer [108], heart failure [109], respiratory distress [110], and gene therapy [111], among others [112,113].

## 7. Conclusions 

The definition of stress has evolved to recognize that responses can vary widely among individual animals and that physiological responses to stress change rapidly over time. Furthermore, repeat exposure of an individual animal to a specific stress may result in adaptation to the stress or facilitation of an enhanced stress response. People involved in animal agriculture now face the challenge of accurately assessing individual animal responses to potentially stressful situations and determining whether homeostasis has been significantly perturbed. In the current review, we emphasized that perturbations of homeostasis have been measured in terms of both altered metabolism and immune function, which then impact animal health, well-being, and productivity. The emerging area of immunometabolism provides a framework for a much more integrated analysis of stress responses. Emerging methodologies, such as RNA-seq and kinome analysis, are species-specific tools that can be used to provide a much more comprehensive analysis of immunometaboism. These technologies provide not only a more comprehensive picture of physiological responses but their high throughput capacity makes it possible to profile dynamic changes that occur over time. The question remaining is how these technologies will be applied to ameliorate the potentially negative effects of animal stress. They will certainly be useful to better define the biological effects of individual or combined stressors and to define the duration of physiological perturbations following exposure to both acute and chronic stressors. A more controversial area may be the application of knowledge provided by these technologies to either the development of stress-inhibiting therapeutics or the genetic selection of animals. Will it be appropriate to ignore stressful animal production or handling systems if pharmacological agents can be used to inhibit physiological responses that negatively impact animal health or production? Alternatively, if selecting animals with a greater tolerance for stressful situations will it then be critical to determine if this selection compromises either animal health or production? The process of animal domestication has been dealing with this later question for thousands of years. We now have the tools, however, to ask more focused questions regarding the needs of both species involved in these mutually beneficial relationships. 
